# Food and Drug Administration Expert Panel on Infant Formula “Operation Stork Speed” June 2025: Part 1, Nutrient Considerations^☆^

**DOI:** 10.1016/j.advnut.2025.100583

**Published:** 2026-01-12

**Authors:** Steven A Abrams, James Thomas Brenna, Roger Clemens, Valeria C Cohran, Nan Du, Andrea Gilbaugh, Michael I Goran, Analeise Guild, John A Kerner, Thomas B Knudsen, Sushma Krishna, Timothy Sentongo

**Affiliations:** 1 Department of Pediatrics, Dell Medical School at the University of Texas, Austin, TX, United States; 2 Department of Regulatory and Quality Sciences, University of Southern California Mann School of Pharmacy and Pharmaceutical Sciences, University of Southern California, Los Angeles, CA, United States; 3 Department of Pediatrics, Ann & Robert H. Lurie Children’s Hospital of Chicago, Northwestern University Feinberg School of Medicine, Chicago, IL, United States; 4 Division of Gastroenterology, Hepatology, and Nutrition, Center for Nutrition, Boston Children’s Hospital, Boston, MA, United States; 5 Clinical Nutrition, Stanford Medicine Children’s Health, Palo Alto, CA, United States; 6 Department of Pediatrics, Children’s Hospital Los Angeles, Keck School of Medicine, University of Southern California, Los Angeles, CA, United States; 7 Global Gastroschisis Foundation, Wake Forest, NC, United States; 8 Division of Gastroenterology, Hepatology, and Nutrition, Division of Neonatology and Developmental Medicine, Stanford University Medical Center, Palo Alto, CA, United States; 9 Department of Intelligent Systems Engineering, Indiana University, Bloomington, IN, United States; 10 Division of Neonatology, NewYork-Presbyterian–Weill Cornell Medicine, New York, NY, United States; 11 Department of Pediatrics, Pritzker School of Medicine, University of Chicago, Chicago, IL, United States

**Keywords:** infant formula, infant nutrition, DHA, lactose, iron, operation stork speed

## Abstract

Operation Stork Speed, launched by the Food and Drug Administration in March 2025, represents a comprehensive initiative to update infant formula regulations that have remained largely unchanged since the 1980s. This expert panel review addresses recommendations for nutrients considering 4 decades of accumulated scientific evidence. Current Food and Drug Administration fatty acid regulations specify only total fat content and minimum linoleic acid requirements, despite substantial international consensus on polyunsaturated fatty acid specifications. Evidence strongly supports establishing maximum linoleic acid concentrations and docosahexaenoic acid and arachidonic acid requirements, reflecting the critical role of omega-3 (ω-3) fatty acids in neurocognitive development and visual acuity. The panel emphasizes that saturated and monounsaturated fatty acids comprise over 80% of human milk fatty acids, while acknowledging recent concerns about seed oils and supporting balanced PUFA formulations. Carbohydrate composition presents significant concerns, as over half of United States formulas contain glucose polymers (e.g., corn syrup solids) despite lactose being the primary carbohydrate energy source in human milk. Observational studies have linked corn syrup-based formulas to multiple potential health risks, including excess weight gain, warranting reconsideration of the value of non-lactose carbohydrate substitutions in formulas for healthy children. Protein content recommendations support decreasing the upper range of allowable intake, aligning with European standards and addressing concerns about excessive protein intake contributing to later obesity risk. Micronutrient evaluation reveals the need to reduce the iron content in routine formulas, consistent with European Food Safety Authority recommendations and emerging safety data, and a need to set upper limits for the concentration of calcium and phosphorus. Overall, infant formula is a healthy product that has been successfully feeding infants for many decades. These comprehensive updates aim to more closely align United States infant formula regulations with current scientific understanding and international standards while supporting optimal infant growth, development, and long-term health outcomes.

**Statements of significance**

This perspective provided information for policy experts in government, industry, and academia regarding a June 2024 expert public panel’s consideration of nutrient requirements for infant formula.

## What Is Operation Stork Speed and What Are Its Goals?

Operation Stork Speed, a joint initiative between the United States Department of Health and Human Services and the United States Food and Drug Administration (FDA) announced in March 2025, sought public input on 4 key areas: nutrient content of infant formulas, testing of heavy metals and contaminants in infant formulas, formula labeling transparency, and identification of scientific research gaps [1]. This paper specifically covers the nutrient content issues, with additional manuscripts covering other related topics from the meeting.

The initiative was launched partly in response to the 2022 infant formula shortages, which exposed supply chain vulnerabilities, and a recent report that raised concerns about potential heavy metal exposure in infant formulas [2]. After introducing Operation Stork Speed and its planned evaluation of the infant formula landscape, the FDA requested that an expert group be convened to discuss key issues and established a plan for a public meeting. The members included pediatricians, nutritional scientists, dietitians, and community members with a special interest in this field. A transcript of this meeting can be found at this site [3].

The goal of this meeting, and of the processes we describe, is to provide guidance to the FDA and other federal officials and government groups, including Congress, on how to support and improve infant formula in the United States. This can be achieved through regulatory and other forms of support that are part of a comprehensive partnership among academia, industry, the community, and government to support the nutritional needs of healthy infants.

## Why Is an Update on Nutrient Requirements in Infant Formulas Needed?

The Infant Formula Act (IFA) of 1980 (Public Law 96-359) established nutrient requirements and safety standards for infant formula and was amended in 1986 (Pub. L. 99-570) to strengthen quality control, manufacturing practices, and regulatory oversight requirements. The IFA serves as the basis for current regulations on infant formula nutrient specifications, which are established in 21 Code of Federal Regulations (CFR) 107.100, originally published 14 January, 1985 (50 Federal Regulations (FR) 1840), with subsequent amendments in 1985 (50 FR 45108), 2015 (80 FR 35841), and 2016 (81 FR 49895), with the 2015 amendment adding selenium requirements to infant formula. No changes have been made to most of the nutrient regulations since 1985.

The IFA is traceable to the events surrounding the chloride deficiency crisis that unfolded in the United States during 1978‒1979. This crisis, which specifically involved 2 soy-based infant formulas, had profound implications for infant health and public trust, which are detailed in a 1984 Congressional Research Service report [4]. Although infant formulas were widely used by the mid-20th century, specific nutrient recommendations were primarily issued by professional organizations, primarily the American Academy of Pediatrics.

In the early 1970s, high sodium intake was identified as a contributing factor to hypertension. Emerging dietary guidelines recommend moderating sodium, including its addition to infant formulas, to prevent infants from developing a reliance on higher sodium concentrations based on taste or physiological response. What was overlooked in this guidance was the requirement for sodium in normal infant metabolism and the coincident intake of chloride. In the well-intentioned desire to reduce sodium, levels were driven so low that some formulas became chloride-deficient.

During 1978 and 1979, reports emerged of infants developing hypochloremic metabolic alkalosis, a serious electrolyte and acid-base imbalance [5]. Investigations quickly linked this condition to the consumption of 2 specific soy-based infant formulas. The manufacturer had reformulated these products by discontinuing the addition of sodium chloride, resulting in formulas with insufficient chloride content to meet the physiological needs of developing infants. The ability of a manufacturer to make such a critical change to an essential nutrient without prior, stringent regulatory review underscored a significant gap in the oversight mechanisms of the time. The clinical manifestations included failure to thrive, characterized by poor weight gain despite adequate caloric intake, diminished appetite, and constipation. Sequelae were also found at 2‒4 y [6] and 9‒10 [7] y of age.

This unfortunate episode is instructive for updating regulations. Extreme recommendations, even when based on well-founded concerns, can have damaging consequences. For example, severely limiting or omitting seed oils in formulas could lead to deficiencies in ω-6 and ω-3 fatty acids, only some of which manifest through overt criteria like poor growth. Neurocognitive development is particularly sensitive, and subtle effects may require expert measurements to detect issues that could magnify later in life.

As new studies on nutrient metabolism appear, specific nutrient concentrations are continuously reevaluated. Manufacturers, interested in distinguishing their products, may view emerging trends as beneficial for business, even if these trends have not been fully vetted for short- or long-term safety. Regular monitoring of requirements based on evolving science is necessary to maintain the highest standards for infant formulas.

## Fats and Fatty Acids

Recent public concern about seed oils has prompted a widespread reconsideration of the edible oil supply. Popular influencers have highlighted 2 major issues: high concentrations of ω-6 linoleic acid (LA) beyond those in pre-industrial foods, and unintended changes in composition during oil refining.

Oils and fats are categorized into 3 groups based on their origin: seed oils, fruit oils, and animal fats. The primary seed oils in the United States, ranked by production volume (in millions of pounds), are soy (11.7), canola/low erucic acid rapeseed (4.7), corn (2.1), sunflower (0.7), cottonseed (0.3), peanut (0.27), safflower (0.2), grapeseed, and rice bran oils [8]. Although high concentrations of ω-6 LA are characteristic of the original forms of these oils, high-oleic varieties with much lower ω-6 LA are widely available for many. High-oleic sunflower oils are the predominant oils from that plant, and high-oleic versions of soy, safflower, and peanut oils are also available. Notably, high-oleic oils have a fatty acid profile like that of olive oil.

Widely available fruit oils are palm oil and its fractions, such as palm olein, coconut, olive, and avocado oils. These oils feature low concentrations of ω-6 LA, substituting it with either MUFAs or SFAs. Apart from extra virgin oils, which are generally cold-pressed, fruit oils are typically processed in a manner like seed oils.

Cow milk fat is the animal fat most relevant to human infant formula. Other possible animal fats are lard (pork rendering) and tallow (beef rendering), both of which require processing. Beyond the fatty acid profiles and the degree of processing, the sourcing of fat is crucial, as all ingredients must consider product uniformity and supply chain stability to meet the annual demand of many metric tons. Overall, seed oils as a category are not distinguished from other oils by either their processing or their ω-6 LA content.

## Fatty Acids Regulations

Current FDA regulations, 21 CFR 107.100, specify only 2 requirements for fat and fatty acids. Total fat must be between 3.3 and 6.0 g/100 kcal (30%‒54% of energy), with the lower range allowed being well below that of human milk, and ω-6 LA must be ≥300 mg/100 kcal of formula, or 2.7% of calories; no maximum amount is specified. These fat and fatty acid requirements have not been updated since their enactment in 1985. The only change in allowable infant formula fatty acid composition was enabled by the FDA in 2001, permitting the addition of single-cell sources of ω-3 DHA and ω-6 arachidonic acid (ARA) to infant formulas. Although the most compelling data for including DHA and ARA in formulas emerged from numerous studies of preterm infants, the no-questions letter allowing use of DHA and ARA applied to term infant formulas as well [9].

Many other countries have updated their specifications, including, for instance, a maximum allowable amount of ω-6 LA and required concentrations of ω-3 DHA and ω-6 ARA [10]. More than a dozen individual and ad hoc groups of pediatric researchers and physicians have published recommendations since the late 1990s for updates on PUFA contents of infant formulas, addressing LA [10,11], ω-3 α-linolenic acid (ALA) [12], ARA [[13], [14], [15], [16], [17]], and DHA [[18], [19], [20], [21]], as well as their relative proportions [[22], [23], [24]]. Consideration of these many treatments has led to a broad consensus on international PUFA regulations for LA, ALA, and DHA concentrations, with some divergence on ARA [10].

## SFAs and MUFAs

SFAs and MUFAs constitute >80% of the total fatty acids (range: 74%‒87%) in human milk [25]. Like all milks, >98% is carried by triacylglycerols (TGs), with most of the balance being phospholipids [26]. Within TGs, palmitic acid is found more prominently, but not exclusively, in the sn-2 position [27], a characteristic of human milk not present in vegetable oils [28]. Lard has palmitic acid in the sn-2 position [29], and cow milk has saturated fats, such as myristic and palmitic acid, predominantly in the sn-2 position [30]. Palmitic acid in the sn-2 position survives digestion in 3-mo-old human infants [28]. Non-esterified SFAs form unabsorbable salts with calcium, leading to the fecal loss of both. On this basis, structured TGs with more palmitic acid (16:0) in the sn-2 position are considered more like those in human milk.

PUFAs are defined as all fatty acids with ≥2 double bonds. The most relevant PUFAs for infant formula are LA, ALA, ARA, and DHA. LA and ARA are ω-6 (n‒6) PUFAs, whereas ALA and DHA are ω-3 (n‒3). Infant formulas with exclusively plant-based oils provide only LA and ALA, requiring the infant’s metabolism to biosynthesize the DHA and ARA that are essential structural components of the brain and all neural tissue. The synthesis and tissue accretion of ARA and DHA proceed with enzymes common to both ω-3 and ω-6 PUFAs [31]. This is the origin of the concept of dietary PUFA balance, most commonly manifested by excess ω-6 LA suppressing ω-3 ALA conversion and creating a metabolic demand for ω-3 long-chain PUFAs (LCPUFAs) [32].

Importantly, SFAs are not vulnerable to attack by reactive oxygen species (ROS), and MUFAs are only minimally affected. In contrast, a key structural feature of PUFAs, the *bis*-allylic position, is the site of oxidation that must be defended from ROS by antioxidants and other metabolic strategies. Thus, SFAs and MUFAs place a minimal oxidative burden on infant metabolism. In contrast, PUFAs in general, and highly unsaturated fatty acids specifically, are highly vulnerable to ROS attack. Consequently, dietary concentrations of PUFA and highly unsaturated fatty acids that meet metabolic requirements without excess are most desirable.

## LA and ALA

Early animal research established that the complete absence of PUFAs in the diet leads to several characteristic deficiency symptoms, specifically skin lesions, loss of water barrier function, polydipsia, and failure to grow. ω-6 LA and ARA were found to be most effective in alleviating these symptoms. Specific studies in human infants established that mild skin lesions, characterized by scaly skin, develop in infants fed formulas with very low PUFA concentrations, a condition that could be reversed by including small amounts of LA [33,34]. Notably, until the 1990s, no pure source of ARA or DHA was available to be safely provided to human infants. In the absence of evidence on ARA and DHA, LA became known as the “essential fatty acid.”

Although subsequent studies show that LA is *metabolically* essential per se [35], not just as a precursor to ARA, definitive studies also show that it is not a *nutritionally* essential PUFA: dietary ARA can be converted to LA to fulfill that metabolic skin function [36]. Mice have been raised on ARA and DHA as the exclusive sources of PUFA through 10 generations with no overt symptoms; at generation 10, neurocognitive development, the function most sensitive to PUFA insufficiency, is normal [37]. LA has persisted as “the essential fatty acid” precisely because of sourcing: the industrial food supply is replete with LA, including oils that are readily available and suitable for use in infant feeds, whereas ARA is a specialty product.

ALA is the ω-3 analog of LA and serves as the precursor for all ω-3 LCPUFAs in diets where no other ω-3 is present. Unlike LA, with its role in skin barrier function, no essential metabolic functions of ALA have been demonstrated. The presence of ALA in the milk of healthy lactating mothers and its role as a nutrient justify its mandatory inclusion in infant formulas.

ALA is available in a small number of seed oils grown at a large scale in North America: soy, canola/rapeseed, and flax. Most oils are deficient in ALA, including sunflower, safflower, corn, peanut, grapeseed, and high-oleic canola. Moreover, fruit oils such as olive, avocado, and palm oils are also deficient in ALA. Olive oil has a reputation for supporting ω-3 concentrations, but this is because it is naturally a low ω-6 LA oil; thus, excess LA above requirements does not suppress ALA conversion or accretion to ω-3 LCPUFAs. Olive oil of typical fatty acid composition is marginally deficient in ω-3.

Before 2001, LA and ALA were the only sources of ω-6 and ω-3 PUFAs in United States infant formulas. These were endogenously converted to ARA and DHA, respectively, to supply tissue demand. Growth, as determined by body weight gain and anthropometrics, matched or exceeded that of breastfed reference infants. However, the early accretion of DHA in the brain [38] led to concerns that DHA synthesis was insufficient in term and especially early preterm infants [39,40].

## DHA and ARA

Neither DHA nor ARA is present in commercial vegetable oils, necessitating the development of specialty oils for infant formulas. Oil from the marine dinoflagellate Crypthecodinium cohnii, commonly referred to as an alga, was the first DHA oil used in United States infant formulas. Schizochytrium oil and egg phospholipids, both generally recognized as safe (GRAS) substances, are also used.

Apart from LA’s function in the skin, DHA and ARA are the bioactive forms of ω-3 and ω-6, respectively. DHA accretion in the neonatal brain accelerates in the last third of term gestation, slows around 2 y of age [40], but continues to 18 y of age [41]. Early human studies used fish oil concentrate-based DHA and EPA, without added ARA, in experimental infant formulas [42], which led to some concerns over ARA-mediated growth [39]. Nearly all subsequent studies included a source of ARA because *Mortierella alpina* oil, a source of ARA, became available. Most of the neurocognitive data ascribed to DHA in infant formulas also contained ARA, and in that sense, their effects on neurocognition apply to the blend of both [13]. The independent role of ARA in immune and vascular function is not well explored. Prudence based on available data suggests that ARA should be included in formulas, though expense remains a serious concern.

**FIGURE 1:**
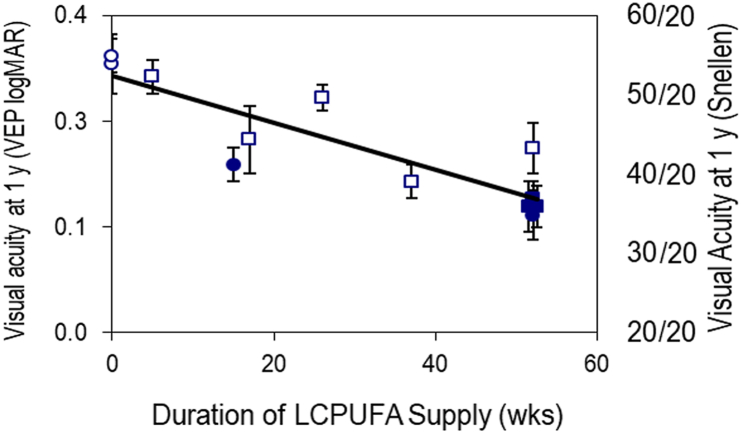
Visual acuity at 52 wk of age as measured by sweep VEP (logMAR). Data are drawn from 4 RCTs in human infants conducted in 1 laboratory and published in 1998–2005. The corresponding Snellen acuities are indicated. Infants who had formula with no LCPUFAs (○) are plotted at 0 wk; others at durations of LCPUFAs from formula or human milk or both (□, ●, ■, as indicated). Values are means ± SEMs; *n* = 15–40/group. Longer exposure to LCPUFAs yielded improved visual acuity at 52 wk of age, reflecting accelerated neural development. LCPUFA, long-chain polyunsaturated fatty acid; logMAR, log of the minimum angle of resolution; RCT, randomized controlled trial; SEM, standard error of the mean; VEP, visually evoked potential. Modified from reference [44] with permission.

Strong evidence for the requirement of DHA and ARA in visual acuity development was established in multiple studies. Visual acuity improves with development largely because of neural development, rather than being restricted to the light-sensing part of the retina. In a series of 4 studies [43], DHA/ARA formulas were compared to formulas with only LA and ALA as sources of PUFA. Figure 1 illustrates visual acuity on the familiar Snellen scale (where 20/20 is normal vision), all measured at 1 y of age. These data show that the longer the exposure to DHA/ARA, the better the vision at 1 y of age [44]. Remarkably, the effect appears whether the DHA/ARA was delivered from a DHA/ARA-supplemented formula or from breastfeeding. Furthermore, these data qualitatively match results from studies in non-human primates investigating ω-3 deficiency [45,46], as well as those using DHA/ARA formulas compared with no-DHA/ARA formulas [47].

## ω-6 for Body, ω-3 for Brain

The foregoing evidence strongly supports consideration of the first update to FDA regulations on fatty acids in infant formulas since they were enacted in 1985. The long interval provides an opportunity to incorporate decades of lessons learned to reset baseline requirements, reflecting both the scientific literature and authoritative recommendations from around the world. Ample data support the use of ω-6 for body growth. Concerns are overwhelmingly about high-priority functions beyond growth. Furthermore, compelling data show that ω-3 DHA supports neurocognitive development, with a focus on balanced fatty acids. Updated regulations should support the development of the whole infant, from head to toe, while providing sufficient flexibility to enable innovation in formulation and giving an expanded range of options for caregivers.

## Carbohydrate Content of Infant Formula

Human milk contains 6% to 8% (g/dL) carbohydrates, of which ∼80% is lactose (a disaccharide of glucose and galactose). Free glucose accounts for only ∼0.1% of human milk, whereas the remaining ∼20% comprises roughly 200 oligosaccharides—non-digestible carbohydrates that act as prebiotics for gut bacteria. Thus, lactose is the sole carbohydrate energy source in human milk.

The IFA does not specify carbohydrate type or set minimum or maximum amounts. The only stipulation is that carbohydrate ingredients, like all others, must be designated GRAS for use in infant formula. Consequently, many United States formulas have replaced some or all of the lactose with other sugars such as glucose-based polymers (corn syrup solids, maltodextrins) and sucrose [48,49], which would be classified as added sugars in other foods. Recent analyses indicate that 59% of powdered formulas sold in the United States contain glucose polymers [48], with the use of non-lactose carbohydrates increasing over time [50].

The reasons manufacturers substitute lactose with other sugars are not clearly defined, but may relate to the cost and solubility advantages of raw ingredients like corn syrup solids compared to lactose. Many lactose-reduced infant formulas are marketed as “gentle” to address possible lactose intolerance in infants. However, lactose intolerance is rare in healthy, full-term infants, as infants are born with the ability to produce the enzyme lactase in the gut, which is needed to break down lactose into glucose and galactose for absorption [51].

Several clinical trials have compared the effects of lactose-based and lactose-reduced formulas in healthy infants [[52], [53], [54]]. These trials generally show that lactose-reduced formulas are safe and support normal growth. Minor differences such as softer stools, less spit-up, and slightly faster weight gain in girls on lactose-free formula were observed, although their significance remains uncertain. Importantly, none of the trials evaluated behavioral, metabolic, or obesity-related outcomes, and all were industry-funded and may have been driven by the high rate of lactose intolerance in adults and older children.

Observational studies, however, suggest potential effects of lactose-reduced formulas compared to human milk or lactose-based formula on behavioral, obesogenic, and metabolic outcomes. Infants fed formulas with corn syrup exhibited steeper increases in food fussiness and reduced enjoyment of food between 12–24 mo [55], as well as higher toddler intake of sugar-sweetened beverages [56]. These feeding behavior changes may contribute to an increased risk of future obesity and/or dysregulated eating styles. In a study of >15,000 infants in California’s special supplemental nutrition program for Women, Infant's and Children (WIC) program, there was a 10% higher risk of obesity at ages 2 and 4 among infants fed corn syrup formulas compared with lactose-based formulas, with a dose-response relationship [57]. For example, 12 mo of exposure to a corn syrup-based formula was associated with a 16% higher risk of obesity by age 2 y. Notably, these effects were independent of maternal weight status, total formula issued, and breastfeeding duration, and were not modified by child race or sex.

Infants fed corn syrup solids or sucrose-based formulas also show disrupted gut microbiota at 6 mo, including lower *Bifidobacteriaceae*, higher *Lachnospiraceae*, and reduced short-chain fatty acids [58]. We currently lack longer-term studies examining whether these gut microbiome shifts persist into childhood and impact clinically relevant outcomes. Theoretical concerns also exist regarding the higher glycemic index of corn syrup formulas, which may lead to elevated blood glucose concentrations, impair healthy glucose homeostasis, and increase pancreatic insulin demand, potentially elevating the later risk for type 2 diabetes—though direct studies in infants are lacking. Importantly, the Dietary Guidelines for America and FDA definitions have not addressed potential glycemic index issues associated with dietary patterns, including the possible effect of soy protein-based formula, physiological status, or behavioral dynamics.

Although these findings are observational and subject to confounding (e.g., parental choice of formula, differing formula compositions), they raise potentially important health concerns. The absence of rigorous randomized controlled trials and longer-term outcomes limits causal inferences and potential public health recommendations. Ethical considerations complicate conducting randomized controlled trials that might expose infants to formulas that could contribute to adverse health effects during critical developmental stages and later in life. Nevertheless, the growing body of evidence showing adverse effects of lactose-reduced and corn syrup-based formulas justifies the need to revisit the rationale and recommendations for replacing lactose with other sugars like corn syrup solids (glucose polymers) and/or sucrose. These other sugars are not present in human milk. They would otherwise be considered added sugars, which, in line with the most recent Dietary Guidelines for Americans (2020‒2025), are not recommended for infants ≤2 y of age.

The other primary carbohydrate source in human milk is human milk oligosaccharides (HMOs). HMOs are a diverse group of complex, indigestible carbohydrates that are the third most abundant component of human milk after lactose and lipids. Built on a lactose core with additional components like fucose and sialic acid, the most abundant HMO in human milk is 2′-fucosyllactose [59]. Though infants cannot digest HMOs, they appear to help shape a healthy gut microbiome, support immune development, protect against infections [60], and are thought to play a role in healthy brain and cognitive development by serving as a source of sialic acid and fucose [61,62]. Some infant formulas now add HMOs, innate to human milk, including 2′-fucosyllactose and lacto-N-neotetraose, both considered GRAS. However, there are no current FDA requirements to include HMOs in formula, and notably, they are not included in formulas designed for premature infants.

We note that this discussion only considers full-term infants, as optimal carbohydrate intake in premature infants requires consideration of a variety of factors, including osmolality and developmental considerations.

## Reassessment of the Recommended Protein Content in Infant Formula for Fealthy Full-Term Infants

The current FDA code of regulations requires a protein content of 1.8 to 4.5 g/100 kcal when its biological value is equivalent to or better than that of casein. If the biological quality is less than casein, the minimum amount of protein must be increased proportionately. This range is significantly higher than the protein content of human milk, which ranges from 1.1 to 1.4 g/100 kcal. Furthermore, not all proteins in human milk have a primary nutritional function; some (e.g., immunoglobulins, lactoferrin) have primary immunological and developmental roles [63].

The primary role of dietary protein is to meet growth and physiological needs by providing adequate amounts of essential and conditionally essential amino acids, as well as total protein. It is also essential to provide adequate protein along with sufficient nonprotein energy sources to ensure that amino acids are not used for energy [64]. Over the past 25 y, significant scientific data have emerged, including: *1*) new infant growth standards (2006) based on exclusively breastfed infants [65]; *2*) the observation that breastfed infants after 6 to 8 wk of life, gain weight more slowly than formula-fed counterparts [65]; *3*) the protective effects of breastfeeding against long-term obesity risk; *4*) emerging evidence linking high protein intake during infancy to later obesity risk [66,67]; and *5*) observations that lower-protein formulas, in the range of human milk, do not increase malnutrition risk [68,69].

In 2005, the European Society of Pediatric Gastroenterology and Nutrition coordinated an International Expert Group that recommended a narrower protein range than 21 CFR 107.100, specific to the protein source: cow milk protein (1.8–3 g/100 kcal), soy protein isolates (2.25–3 g/100 kcal), and hydrolyzed cow milk protein (1.8–3 g/100 kcal) [70]. Additional pertinent data since 1998 include the introduction of 2 new growth charts: the Centers for Disease Control and Prevention 2000 growth reference [71] and the 2006 WHO/Centers for Disease Control and Prevention growth standard for children <24 mo [65]. The 2006 standard, based on longitudinally collected data from healthy, exclusively breastfed infants, provides a prescription for how healthy infants should grow and highlights significant growth pattern differences compared with formula-fed infants.

As a group, formula-fed infants often gain weight, especially after the first 6 to 8 wk of life, more rapidly than their breastfed counterparts [65]. Rapid weight gain during infancy has been associated with an increased risk of adiposity, overweight, and obesity [69,72]. Epidemiological studies suggest a link between higher-protein intake in early life and a greater risk of overweight and obesity in children and adolescents [66]. Clinical trials comparing lower-protein (1.77 g/100 kcal) with higher-protein (2.9 g/100 kcal) formulas have shown that infants on lower-protein formulas exhibit growth patterns similar to breastfed infants and slower rates of weight gain than those on higher-protein formulas [69].

Therefore, the 1998 broad range for protein content may benefit from being narrowed, for example, to 1.8 to 3 g/100 kcal/d. This aligns with ranges associated with normal growth, decreased long-term risk of overweight and obesity [73], and harmony with European Society of Pediatric Gastroenterology and Nutrition – International Expert Group recommendations [70].

## Protein Quantity

Protein quality is a consideration for infant formula protein content. The IFA minimum and maximum concentrations for protein are 1.8 g/100 kcal to 4.5 g/100 kcal, which are consistent with those supported by the CODEX Alimentarius standard [74] and Canadian regulations [75]. However, a commentary by Liotto [76] suggested that high protein intake by infants, relative to human milk, may alter growth rates and possibly contribute to an increased risk of adiposity and affect renal function. A study of full-term infants in Europe and a subsequent systematic review suggest that protein intake on the lower end or just below the allowable range (∼1.7‒1.8 g/100 kcal) may promote more efficient protein use and a preferred fat-free mass profile, while supporting normal growth [77,78].

## Amino Acid Profiles

Infant formulas in the United States typically consist of bovine casein and whey blends, which are analogous to proteins in human milk. However, the physicochemical properties of the human milk proteins differ from those of bovine sources. Genetic variants affecting these properties require consideration when formulating infant formula [79,80]. These differences are also apparent in soy protein concentrates/isolates. The respective amino acid profiles and protein quality can influence critical factors such as infant growth and development.

Table 1 depicts amino acid profile differences among bovine casein (≥4 types of casein, most of which are A1 and A2 β-casein), bovine whey, which includes β-lactoglobulin, α-lactalbumin, immunoglobulins, and bovine serum albumin, and typical soy protein isolate (often differs in physicochemical properties). Although bovine casein, whey, and soy protein isolates dominate United States infant formulas, their amino acid profiles are different than that of human milk. These differences support the need for evaluation of novel protein blends that may improve the amino acid profile and lead to better growth and development outcomes, as well as possibly reduce risks for noncommunicable diseases [81,82]. The use of free amino acids may also be considered as a strategy to affect protein composition, yet their inclusion will increase the osmolarity of infant formula and may create sensory or regulatory challenges.

**TABLE 1:** Dynamics of amino acid profiles.

Source	His	Ile	Leu	Lys	SAA			AAA	Thr	Trp	Val
					Total	Met	Cys				
(A) Bovine casein	26	90	51	75	28	25	3	100	41	13	61
(B) Bovine whey	16	74	121	109	52	25	27	75	88	17	69
(C) Soy protein isolate	27	43	78	65	28	14	14	95	36	10	45
(D) Human milk	23	51	94	63	35	14	21	87	43	18	50
Ratio A:D	1.13	1.76	0.54	1.19	0.80	1.79	0.14	1.15	0.95	0.72	1.22
Ratio B:D	0.70	1.45	1.29	1.73	1.49	1.79	1.29	0.86	2.05	0.94	1.38
Ratio C:D	1.17	0.84	0.83	1.03	0.80	1.00	0.67	1.09	0.84	0.56	0.90

Data presented as milligram amino acids per gram of protein; bolded font indicates amino acid concentrations less than those typically found in mature human milk.

Abbreviations: AAA, aromatic amino acid; Cys, cysteine; His, histidine; Ile, isoleucine; Leu, leucine; Lys, lysine; Met, methionine; SAA, sulfur amino acid; Thr, threonine; Trp, tryptophan; Val, valine.

## Protein Quality

In 1994, the FDA adopted the protein digestibility-corrected amino acid score (PDCAAS) method instead of the protein efficiency ratio (PER) to assess protein quality for conventional foods, but not for infant formula [83]. The contention was that the PER method, based on Animal Nutrition Research Council (ANRC) casein, represented the protein requirements for humans when assessed in a rodent model. A recent perspective notes that nonprotein nitrogen was not considered in the PER method, which therefore did not reflect the amino acid availability and protein digestibility of human milk [84]. When ANRC casein was no longer available, the industry and Association of Official Analytical Collaboration (AOAC) International conducted studies to qualify alternative casein standards. In 2023, the FDA announced a draft update for conducting PER studies [85], and AOAC International published an update on the PER method [86].

With respect to PDCAAS, protein digestibility is variable [87]. Furthermore, when evaluating protein hydrolysates via PDCAAS, the pre-existing hydrolysis (a process to reduce allergenicity) suggests a lower-protein quality than expected [88]. This is essential as the international community transitions to the digestible indispensable amino acid score, advanced by the FAO in 2013 to replace PDCAAS. This approach is under consideration by the FDA and Health Canada and is considered an improved method for assessing protein quality in complex food matrices [89]. However, the digestible indispensable amino acid score is not without limitations, particularly concerning plant-based diets and the search for new alternative methods to reduce animal testing [89,90].

## Micronutrient Considerations in Infant Formula Regulatory Review

Guidance for most micronutrients, such as iron, bone minerals, and trace minerals (except selenium), was set pursuant to the 1980 IFA. Many of these values were based on limited experimental data and were primarily established through estimates of physiological requirements and human milk intake. A large body of evidence has since accumulated, making it appropriate to reevaluate these values and establish maximum intake levels where none existed before. This includes establishing maximum concentrations for calcium and phosphorus.

Of most significant importance is the re-evaluation of guidance related to the iron content of infant formulas. As we have recently reviewed in detail, and as also reviewed by the European Food Safety Authority (EFSA) and others, available data suggest that the maximum iron allowed in United States infant formula (3.0 mg/100 kcal, 20 mg/L) is too high [[91], [92], [93], [94]]. The usual intake in nearly all United States formulas (12 mg/L, 1.8 mg/100 kcal) is far above the maximum set by EFSA of 1.3 mg/100 kcal and is at a level where substantial data suggest a greater risk of harm than benefit. The United States maximum should be reduced to no >10 mg/L (∼1.5 mg/100 kcal), and consideration should be given to matching the EFSA limit of 8.7 mg/L (1.3 mg/100 kcal) [91], but also to indicate that this value should be the average of analyzed samples, not the lowest found in any sample. This would reduce the need for companies to have an overage during production while targeting 8 mg/L as a usual label goal.

The IFA does not specify maximum values for calcium or phosphorus in infant formulas. In the United States, the minimum calcium is 60 mg/100 kcal, and the minimum phosphorus is 30 mg/100 kcal. The molar calcium to phosphorus ratio must be not <1.1 and not >2.0. In contrast, human milk calcium is 31 to 46/100 kcal. The molar ratio of calcium to phosphorus is ∼1.6:1. Although EFSA also does not set a maximum for these nutrients, they are set by CODEX, which has a calcium maximum of 140 mg/100 kcal and a phosphorus maximum of 100 mg/100 kcal.

Although calcium bioavailability is likely higher in human milk than in formula, this has not been tested at the same concentration, and actual differences may be minor. This is especially important as human milk phosphorus is often 15 to 20 mg/100 kcal, and this low level is protective against late hypocalcemic tetany. Overall, it is likely that although there is no substantial risk due to excess calcium or phosphorus in infant formula, the CODEX guidance to set maximum levels for these, alongside the ratio of the 2, should be considered, especially given their use in smaller full-term infants and early in life.

In conclusion, the nutritional value of infant formula is dependent on an array of components, including fat, carbohydrate, protein, and micronutrients. Nearly 30 y ago, the infant formula industry added DHA and ARA to their products, yet the FDA has yet to establish minimum and maximum concentrations of these fatty acids. Regardless, the industry has attempted to mimic the ratio of these fatty acids relative to human milk composition while relying on emerging scientific evidence related to health benefits. Regarding carbohydrate moieties, lactose is the dominate sugar in human milk, followed by HMOs. However, most infant formulas contain other carbohydrates, such as corn syrup solids, sucrose, and maltodextrins, which are not present in breastmilk, and emerging evidence indicates adverse effects on infant outcomes, including increased risk for obesity.

More than 20 y ago, the formula industry introduced oligosaccharides that are found in human milk to potentially improve the immune function and gut flora of infants. Although limited in number, these may be important for infant health. Those included represent a small proportion of the over 200 unique carbohydrates in human milk. More research is needed to identify the optimal mix of these oligosaccharides to optimize health outcomes. However, they are currently not required by regulation and are not found, for example, in many formulas such as those used by government programs. In terms of micronutrients, these should be reassessed and consideration given to align standards more closely with global standards, especially for iron.

Overall, infant formula is a healthy product and has been successfully feeding infants for many decades. Nutrient-related updates in regulatory guidance can improve the products, consistent with the latest science.

## Author contributions

The authors’ responsibilities were as follows – All authors: read and approved the final manuscript.

## Funding

JTB has received honoraria or travel funds from the Global Organization for EPA and DHA, the Global Dairy Platform, and Danone, as well as research support from the National Cattlemen’s Beef Association. The authors have no additional financial support to declare.

## Conflict of interest

SAA is editor-in-chief of Advances in Nutrition and played no role in the journal’s evaluation of the manuscript. MIG has previously served as a scientific advisor for Begin Health and Bobbi. VCC has served on the Speaker’s Bureau for Nutricia and Abbott Nutrition. All other authors report no conflicts of interest.
